# Effect of Sperm DNA Fragmentation on Clinical Outcome of Frozen-Thawed Embryo Transfer and on Blastocyst Formation

**DOI:** 10.1371/journal.pone.0094956

**Published:** 2014-04-14

**Authors:** Wuhua Ni, Shiquan Xiao, Xiufang Qiu, Jianyuan Jin, Chengshuang Pan, Yan Li, Qianjin Fei, Xu Yang, Liya Zhang, Xuefeng Huang

**Affiliations:** Reproductive Medical Center, The First Affiliated Hospital of Wenzhou Medical University, Wenzhou, Zhejiang, China; University Hospital of Münster, Germany

## Abstract

During the last decades, many studies have shown the possible influence of sperm DNA fragmentation on assisted reproductive technique outcomes. However, little is known about the impact of sperm DNA fragmentation on the clinical outcome of frozen-thawed embryo transfer (FET) from cycles of conventional in vitro fertilization (IVF) and intra-cytoplasmic sperm injection (ICSI). In the present study, the relationship between sperm DNA fragmentation (SDF) and FET clinical outcomes in IVF and ICSI cycles was analyzed. A total of 1082 FET cycles with cleavage stage embryos (C-FET) (855 from IVF and 227 from ICSI) and 653 frozen-thawed blastocyst transfer cycles (B-FET) (525 from IVF and 128 from ICSI) were included. There was no significant change in clinical pregnancy, biochemical pregnancy and miscarriage rates in the group with a SDF >30% compared with the group with a SDF ≤30% in IVF and ICSI cycles with C-FET or B-FET. Also, there was no significant impact on the FET clinic outcome in IVF and ICSI when different values of SDF (such as 10%, 20%, 25%, 35%, and 40%) were taken as proposed threshold levels. However, the blastulation rates were significantly higher in the SDF ≤30% group in ICSI cycle. Taken together, our data show that sperm DNA fragmentation measured by Sperm Chromatin Dispersion (SCD) test is not associated with clinical outcome of FET in IVF and ICSI. Nonetheless, SDF is related to the blastocyst formation in ICSI cycles.

## Introduction

Sperm DNA damage is increasingly being recognized as an important cause of infertility and has better diagnostic and prognostic capabilities than routine semen parameters [1]. Routine semen parameters may not reveal sperm defects affecting the integrity of the male genome. One of the main cause of male infertility with normal spermiogram may be related abnormalities in the male genome characterized by damaged DNA, which is highly indicative of male subfertility regardless of routine semen parameters [2]–[4].

Assisted reproductive technique (ART) has revolutionized the management of severe male infertility and increased the chance of sperm with abnormal genome to fertilize the oocyte [1]. In the last decades, many studies have shown the possible influence of sperm DNA damage on ART outcomes [5]–[14]. Sperm DNA damage has been shown to adversely affect reproductive outcomes [15], although the true clinical significance of sperm DNA damage assays remains to be established since the available studies are few and heterogeneous [16]. A recent systematic review showed that sperm DNA damage is associated with lower pregnancy rate of natural, intrauterine insemination (IUI), and in vitro fertilization (IVF), and an increased risk of pregnancy loss in those couples undergoing IVF or intracytoplasmic sperm injection (ICSI). Sperm DNA damage is however, not associated with pregnancy rate by ICSI [16].

Frozen-thawed embryo transfer (FET) has become a vital component of ART[17]. Chance of pregnancy following FET treatment has usually been lower than that of fresh embryo transfer [18]. Nevertheless, FET is a cost-effective and less invasive procedure, which can be accomplished in a shorter time period compared with repeated “fresh” cycles, and increases the cumulative pregnancy rate [18]. A recent study [19] has shown that poor sperm quality as indicated by routine sperm parameters affects clinical outcomes of the subsequent FET cycles following ICSI. However, little is known about the impact of sperm DNA fragmentation on clinical outcome of FET following conventional IVF and ICSI. In the current study, we sought to evaluate the relationship between sperm DNA fragmentation and FET outcome after standard IVF and ICSI.

## Materials and Methods

### Patients

The study was based on a cohort of consecutive infertile couples undergoing FET at the First Affiliated Hospital of Wenzhou Medical University during the period of April 2009 to March 2012. A total of 1082 FET cycles with cleavage stage embryos (C-FET) (855 from IVF and 227 from ICSI) and 653 frozen-thawed blastocyst transfer cycles (B-FET) (525 from IVF and 128 from ICSI) were included. Male partners had a sperm concentration of at least 1×10^6^/ml in raw semen. Inclusion criteria were women with no known gynecological pathology (e.g., known endometriosis, fibroids, any previous operation to gynecological organs) except for tubal factors. FET cycles from the fresh cycles with less than 5 oocytes were excluded in this study.

### Ethics statement

The study was approved by the Medical Ethics Committee of The First Affiliated Hospital of Wenzhou Medical University, and written consent was obtained from all study patients. The consent procedure was approved by the Medical Ethics Committee of The First Affiliated Hospital of Wenzhou Medical University.

### Sperm DNA fragmentation analysis

The principles and procedure of measuring sperm DNA fragmentation by Sperm Chromatin Dispersion (SCD) test are described in detail [20]. Briefly, SCD test was performed using the Halosperm kit (INDAS Laboratories, Madrid, Spain) according to the manufacturer's protocol. The SCD test is based on the principle that sperm with fragmented DNA fail to produce the characteristic halo of dispersed DNA loops that is observed in sperm with non-fragmented DNA, following acid denaturation and removal of nuclear proteins [21]. The extent of DNA damage for each semen sample is expressed as the sperm DNA fragmentation index (SDF). In humans, a threshold of 30% SDF is frequently suggested as a cut-off to distinguish between a potentially fertile *vs* infertile semen sample, thus all men were classified into two groups regarding the SDF threshold value: ≤30% and >30%.

### ART procedures

Ovarian stimulation was performed using standard leuteal down-regulation regimen or flare-up short regimen. The standard IVF or ICSI technique was used to inseminate the retrieved oocytes. After 16–18 hrs of insemination, the oocytes were assessed to determine whether fertilization had occurred. The cleavage embryos on day 2 or day 3 were graded according to their morphology and developmental speed. Fresh embryo transfer was performed on day 2 or day 3 after oocyte retrieval using the best quality embryos among a cohort of resultant embryos. The surplus embryos were cryopreserved in day 3 or in day 5–6 according to patient's requirement. The day 3 embryos eligible for cryopreservation were those with less than 30% fragmentation and 6–10 blastomeres. The day 5–6 blastocyst that were cryopreserved had at least grade 3BB. Freezing and thawing were performed using the ADVITRO Vitrification Freeze and Thaw kit (Shanghai disease control and biological technology co., LTD, shanghai, China) according to the manufacturer's protocols. Briefly, embryos were first incubated in ES(Equilibration Solution, HEPES-buffered medium, 7.5% (v/v) of DMSO and ethylene glycol and 20% (v/v) serum protein substitute.) for 5 minutes and then transferred to VS (Vitrification Solutions, HEPES-buffered medium, 15% (v/v) of DMSO and ethylene glycol and 20% (v/v) serum protein substitute and 0.5 mol/L sucrose). The embryos were then loaded into a sterile straw (Cryoleaf) with a minimal volume, and then the straw was quickly immersed into liquid nitrogen. For thawing of the frozen human embryos, the straws was immersed directly into a 37°C TS (Thawing Solution, HEPES-buffered medium, 1.0 mol/L sucrose and 20% (v/v) serum protein substitute) for 1 minute. The embryos were then transferred to DS (Diluent Solution, HEPES-buffered medium, 0.5 mol/L sucrose and 20% (v/v) serum protein substitute) for 3 minutes, then into WS (Washing Solution, HEPES-buffered medium and 20% (v/v) serum protein substitute) for 5 minutes and then into another WS for 5 minutes. The thawed embryos were cultured in a humidified atmosphere of 5% CO_2_ in air, at 37°C.

### Replacement Preparation and Assessment of Pregnancy

Transfer of frozen–thawed embryos was performed either in natural or in hormone replacement cycle. No more than 3 survival embryos were transferred into the uterine cavity. The luteal phase was routinely supported with progesterone 40–60 mg IM per day for 14 days and continued for another 4 weeks if pregnancy was established. Serum HCG was checked 2 weeks after ET, and ultrasound was further performed to confirm if there are any intrauterine gestational sacs 4 weeks after ET. A clinical pregnancy is defined as positive HCG with intrauterine gestational sac, while a biochemical pregnancy is defined as positive HCG without any intrauterine gestational sac. To calculate the implantation rate, the number of gestational sacs was divided by the number of embryos transferred. Miscarriage was defined as spontaneous abortion before 20 weeks of gestation. The live birth rate is the percentage of transfers that lead to a live birth.

### Statistical analysis

Data were analyzed using the SPSS 17.0 for Windows (SPSS Inc., Chicago, USA). The Student's t-test for independent samples (SPSS) was used for comparison of means. The chi-square test was used for group comparison of good embryo rate, embryo post-thaw survival rate, implantation rate, biochemical and clinical pregnancy rates, and miscarriage rate. Differences were considered statistically significant at *P*<0.05.

## Results

### Clinical parameters and outcome of C-FET

A total of 1082 FET cycles with cleavage stage embryos (C-FET) (855 from IVF and 227 from ICSI) were retrospectively analyzed in this study. According to sources of the frozen-thawed embryos, all C-FET cycles were divided into group IVF or group ICSI. The two groups were further subdivided into two sub-groups according to the level of SDF (SDF ≤30% and >30%). In Table 1, the clinical data of the two groups are reported in IVF and ICSI cycles with C-FET. The two groups were homogeneous for female age, male age, number of oocytes retrieved, number of oocytes fertilized, number of embryos frozen, frozen time and implantation rate. Semen samples that had a higher DNA damage (SDF >30%) showed a lower concentration in ICSI group and lower motility in IVF and ICSI group.

**Table 1:** Clinical data on frozen-thawed embryo transfer with cleavage stage embryos (C-FET) cycles divided according to the type of treatment; IVF and ICSI.

SDF	IVF		ICSI	
	≤30%	>30%	≤30%	>30%
Cycles included (n)	783	72	127	100
Female age(years± SD)	31.4±3.8	32.2±4.3	31.6±4.0	31.1±3.2
Oocytes retrieved(n± SD)	15.1±6.0	13.2±6.1	16.7±7.2	14.4±5.6
Oocytes fertilized(n ± SD)	9.8±4.1	9.6±4.2	10.6±5.0	9.4±3.6
Embryos frozen(n± SD)	5.9±3.4	5.2±3.4	7.2±4.4	6.1±3.2
Frozen time(day± SD)	114.0±165.9	88.8±72.7	96.2±142.9	120.9±171.7
Male age (years± SD)	33.5±4.3	34.9±4.5	33.2±4.3	34.1±4.2
Embryo transplant(n± SD)	2.4±0.7	2.4±0.7	2.4±0.7	2.3±0.8
SDF	14.8±6.3^b^	41.2±8.8	18.2±6.3^b^	47.3±15.2
Sperm concentration(×10^6^/ml)	96.1±63.7	73.1±51.2	53.3± 61.3^b^	14.0±24.8
Sperm motility (%)	63.2±12.4^a^	45.1±16.2	40.4±24.9^b^	25.3±18.6
Sperm morphology	8.6±4.0	6.9±4.4	6.0±4.0	4.6±3.7
Implantation rate	9.5% (182/1914)	10.1% (17/168)	10.3% (32/311)	12.3% (29/235)

a *P*<0.05, ^b^ *P*<0.01 for comparison with the SDF >30% group.

There was no significant change of obtaining clinical pregnancy, biochemical pregnancy, live birth and miscarriage rates in the group with a SDF >30% compared with the group with a SDF ≤30% in IVF and ICSI cycles with C-FET, although it is worth noting that the miscarriage rate is higher in SDF >30% group in IVF and ICSI (Table 2). In addition, the effect of varying the SDF threshold value (such as 10%, 20%, 25%, 35%, and 40%) to predict pregnancy outcome was analyzed in IVF and ICSI cycles. No significant change in effect was found when different values of SDF were taken as proposed threshold levels in IVF and ICSI with C-FET.

**Table 2:** Data on pregnancy and miscarriage rates in 855 IVF and 227 ICSI cycles with C-FET divided according to SDF ≤30% versus SDF >30%.

SDF	IVF		ICSI	
	≤30%	>30%	≤30%	>30%
Cycles included (n)	783	72	127	100
Biochemical pregnancy rate	4.9%(38/783)	6.9%(5/72)	5.5% (7/127)	5% (5/100)
Clinical pregnancy rate	21.1%(165/783)	23.6% (17/72)	22.0% (28/127)	26.0% (26/100)
Live birth rate	15.7%(123/783)	16.7%(12/72)	16.5%(21/127)	16.0%(16/100)
Miscarriage rate	18.8%(31/165)	23.5% (4/17)	17.9% (5/28)	30.8% (8/26)

### Clinical parameters and outcome of B-FET

A total of 653 frozen-thawed blastocyst transfer cycles (B-FET) (525 from IVF and 128 from ICSI) were analyzed. Patient groups were similar in regards to their age, number of oocytes retrieved, number of oocytes fertilized, number of embryos frozen, frozen time and implantation rate (Table 3). The sperm concentration in ICSI group and sperm motility in IVF and ICSI group were significantly lower in men with SDF >30%.

**Table 3:** Clinical data on frozen-thawed blastocysts transfer cycles divided according to the type of treatment; IVF and ICSI.

SDF	IVF		ICSI	
	≤30%	>30%	≤30%	>30%
Cycles included (n)	503	22	88	40
Female age(years± SD)	31.4±3.9	32.5±3.9	30.9±4.6	30.6±4.0
Male age(years± SD)	33.5±4.5	35.8±5.6	33.9±4.9	33.8±3.8
Oocytes retrieved(n± SD)	13.6±6.2	15.8±7.5	13.0±5.8	13.6±4.7
Oocytes fertilized(n ± SD)	9.5±4.3	11.6±4.2	8.9±4.3	8.6±3.3
Embryos frozen(n± SD)	3.2±2.2	3.9±3.7	3.02±1.88	2.23±1.31
Embryo transplant(n± SD)	1.8±0.5	1.9±0.5	1.7±0.5	1.8±0.4
SDF	12.4±6.4^b^	40.9±11.6	16.3±7.1^b^	43.5±12.4
Sperm concentration(×10^6^/ml)	92.8±68.2	51.4±73.1	58.0±58.9^b^	19.6±37.2
Sperm motility (%)	61.0±15.7^b^	34.1±19.4	47.9±21.6^a^	27.1±18.6
Sperm morphology	8.5±3.5	7.2±4.7	6.7±4.3	4.3±3.4
Implantation rate	35.58(306/860)	30.76(12/39)	28.76(42/146)	32.31(21/65)

a *P*<0.05, ^b^ *P*<0.01 for comparison with the SDF >30% group.

The outcomes of B-FET are detailed in table 4. The clinical pregnancy, biochemical pregnancy, live birth and miscarriage rates showed no significant differences in the group with a SDF >30% and the group with a SDF ≤30% in IVF and ICSI cycles. In addition, no statistically significant difference was found in the blastulation rates between the two groups in IVF cycles. However, the blastulation rates were significantly higher in SDF ≤30% group in the ICSI cycle. When different values of SDF (such as 10%, 20%, 25%, 35%, 40%) were taken as proposed threshold levels in IVF and ICSI, there was no significant difference in blastulation, clinical pregnancy, biochemical pregnancy, live birth and miscarriage rates between high and low SDF group.

**Table 4:** Data on blastulation, pregnancy, implantation and miscarriage rates in 525 IVF and 128 ICSI cycles divided according to SDF ≤30% versus SDF >30%.

SDF	IVF		ICSI	
	≤30%	>30%	≤30%	>30%
Cycles included (n)	503	22	88	40
Blastulation rate	44.17%(1629/3697)	41.7%(85/204)	45.5%(266/584)^a^	34.9%(89/255)
Biochemical pregnancy rate	7.2%(36/503)	0%(0/22)	10.2%(9/88)	2.5%(1/40)
Clinical pregnancy rate	49.3%(248/503)	50%(11/22)	43.2%(38/88)	45%(18/40)
Live birth rate	37.2%(187/503)	40.9%(9/22)	31.8%(28/88)	35.0%(14/40)
Miscarriage rate	14.9%(37/248)	9.1%(1/11)	10.5%(4/38)	16.7%(3/18)

a *P*<0.01 for comparison with the SDF >30% group in ICSI.

## Discussion

Animal studies have shown that mammalian fertilization and subsequent embryo development depend in part on the inherent integrity of the sperm DNA and that there appears to be a threshold of sperm DNA damage (e.g. DNA fragmentation) beyond which embryo development and pregnancy are impaired [22]. There is now clinical evidence to suggest that damage to human sperm DNA may adversely affect reproductive outcomes [15]. In addition, spermatozoa of infertile men possess substantially more DNA damage than spermatozoa of fertile men [15]. Conventional semen analysis by assessing sperm concentration, motility and morphology are not able to assess alterations in sperm chromatin organization, such as irregular condensation or DNA damage [23]. Both direct (fragmentation, oxidation) or indirect (sperm chromatin compaction) tests of sperm DNA damage are now available [15]. These include TUNEL assay, “Comet” assay, chromomycin A3 test, DNA Breakage Detection-Fluorescence In Situ Hybridization (DBD-FISH), Sperm Chromatin Structure Assay (SCSA) and Sperm Chromatin Dispersion (SCD) test [21]. The SCSA is the current commonly used standard for the quantitative determination of DNA fragmentation and has been reported mostly [21], [24]. However, the SCSA is expensive, time-consuming and requires complex equipment, not accessible to most andrology laboratories [24]. Sperm DNA denaturation as measured by the SCD test, correlates strongly with other markers of DNA damage such as DBD-FISH analysis and SCSA [21], [25]. Unlike the SCSA, the SCD test can be used without the requirement of complex or expensive instrumentation [26]. Also unlike the TUNEL assay, the comet assay, and the chromomycin A3 test for the determination of sperm DNA fragmentation, the SCD test does not rely on the determination of either color or fluorescence intensity [21]. In addition, laboratory technicians can quickly and reliably assess the test end points, which consists of the percentage of spermatozoa with no dispersed (very small halos or none at all) or dispersed nuclei, using a light microscope [21]. Therefore, the SCD test could potentially be used as a routine sperm DNA fragmentation test in a clinical andrology laboratory [21].

In our study, the SCD test was used to evaluate the sperm DNA fragmentation. Sperm DNA fragmentation has been associated with reduced fertilization rate, embryo quality and pregnancy rate, and increased incidence of spontaneous miscarriage [27]. However, very little is currently known regarding the impact of sperm DNA fragmentation on clinical outcome of FET. Embryo cryopreservation is a well-established technique that allows the storage of supernumerary embryos created during ART for later transfer [28]. The outcome of FET cycles is affected by several factors including maternal age, number of embryos transferred, and possible differences in frozen-thawed protocols and culture conditions [29], [30]. Also, the factors associated with the fresh cycle, namely the total number of oocytes collected, and quality of embryos available for cryopreservation [31], [32] have been shown to have an impact on the corresponding FET cycle. In this study, there are no differences in those variables among the thaw cycles of the analyzed groups.

Sperm characteristics may be one of the factors to influence clinical result of FET. Viability of each embryo transferred depends on the biological quality of the oocyte and the spermatozoon at the given embryo's origin [33]. The paternal contributions to early embryo development have been shown to be responsible for repeated failures of assisted reproduction attempts [8], [34]. The FETs from epididymal and testicular sperm have been shown to have similar clinical results [33]. The abnormalities of sperm parameter, however, have been shown to have negative impact on the clinical result of FET [19]. The rates of implantation and clinical pregnancy of normal-spermatogenesis patients (NSPs) were significantly higher than those of defective-spermatogenesis patients (DSPs) [19]. To the best of our knowledge, results of the present study point first to the impact of sperm DNA fragmentation on clinical outcome of FET. Sperm DNA fragmentation is associated with longer times to conceive [35], impaired embryo cleavage [7], higher miscarriage rates [36], and a significantly increased risk of pregnancy loss after IVF and ICSI compared with fertile couples [37]. In this study, the clinical pregnancy, biochemical pregnancy and miscarriage rates showed no statistical difference between SDF ≤30% group and SDF >30% group in IVF and ICSI cycles with C-FET or B-FET. A reason for the current finding may be due to the fact that detected sperm DNA fragmentation in raw semen may not exactly reflect the quality of selected sperm applied in FET. The assays for sperm DNA fragmentation were performed on raw semen samples that maybe contain a high percentage of immotile, nonviable or degenerated sperm with abnormal chromatin. However, most abnormal sperms were removed through gradient centrifugation, swim up or glass wool techniques before the selected sperms were applied in ICSI or IVF [38], [39]. Another reason for the current finding could be that embryos have been selected before being frozen because the surplus embryos suitable for freezing usually come from the IVF or ICSI cycles with better quality embryos. In addition, sperm DNA integrity does not represent all of the paternal effects controlling early embryonic activities after IVF/ICSI treatment. Although we did not observe a significant difference in the risk of miscarriage between SDF ≤30% group and SDF >30% group in ICSI cycles with C-FET or B-FET, the miscarriage rates increased from 17.9% (5/28) to 30.8% (8/26) in ICSI cycles with C-FET and from 10.5% (4/38) to 16.7% (3/18) in ICSI cycles with B-FET. The powers of this analysis were too low (less than 0.80) to claim that no difference exists between these two groups and a larger sample size is needed.

Moreover, our data showed that the blastulation rates were significantly higher in the SDF ≤30% group in ICSI cycle. This result is consisted with previous report [9], [40]. A high level of DNA fragmentation (SDF >30%) in sperm cells may be with greater risk for blastocyst formation [9]. Nasr-Esfahani MH et al. reported that embryos derived from spermatozoa with high DNA damage have a lower potential to reach later or blastocyst stage [40]. However, blastulation rates were no different between the group with a SDF >30% and the group with a SDF ≤30% in IVF cycles. The most likely explanation for this is the natural selection during IVF. There is a close relationship between sperm DNA integrity, sperm motility and sperm membrane characteristics (with the latter characteristic being important for sperm-cumulus and sperm-zona binding). In theory, while using IVF, the probability of fertilization with DNA-damaged sperm should be reduced by natural selection processes [41]–[44]. A number of studies have been conducted to examine the possible influence of sperm DNA damage on reproductive outcomes after both standard IVF and IVF/ICSI, showing no consistent relationship[2], [7]–[9], [12], [15], [37], [41], [45]–[48]. Morris el al. demonstrated that sperm carrying high DNA damage measured by the Comet assay does not adversely affect the implantation and pregnancy outcome after ICSI [7]. In this study, the implantation and pregnancy outcome were not significantly different in the group with a SDF >30% and the group with a SDF ≤30% in ICSI cycles, although the blastulation rates were significantly higher in SDF ≤30% group. However, the size of this study does not allow us to make the conclusion that the presence of sperm DNA damage doesn't adversely affect the pregnancy outcome in B-FET. Blastocyst FET may undergo self-selection processes twice. One selection is the avoidance of arrest through the extended culture and another is the survival during the freezing-thawing processes [49]. During ICSI, the fertilizing sperm is randomly picked and it is possible that sperm possessing damaged DNA will be selected and used to fertilize oocytes [40], [50]. Theoretically, blastocyst culture induces self-selection of viable embryos through the period of extended culture [51], and only those zygotes with a relatively intact genome could develop into blastocysts [7], [40]. However, it still remains a chance that low, sublethal levels of sperm DNA damage are transmitted to embryos [7], [40], [50]. Such low levels of DNA damage may be insufficient to cause a gross response such as cell cycle arrest or apoptosis prior to implantation, or early pregnancy failure, but may nonetheless be expressed during fetal or post-natal development [7], [40], [50]. Moreover, the biological impact of sperm DNA damage depends on the combined effects of the level of sperm DNA damage and the capacity of the oocyte to repair that damage [52], [53].

However, IVF and ICSI cannot overcome abnormalities in DNA integrity and will bypass the natural selection of normal, healthy sperm and may lead to fertilization by sperm with damaged DNA. As sperm have few repair mechanisms [54] and oocytes can only repair a limited amount of sperm DNA damage [55], [56], the damage may remain unrepaired or be aberrantly repaired, causing DNA mutations in the germ line for generations. DNA-damaged sperm has the ability to fertilize the oocyte and damaged DNA may be incorporated into the embryonic genome. During embryogenesis, DNA damage leads to errors in DNA replication, transcription and translation, contributing to a range of human diseases [57] in not just one but subsequent future generations [58]. In particular, sperm DNA can impact the short and long term health of children conceived by ART [12]. Sperm DNA damage appears as a risk factor for an elevated risk of morbidity in the offspring [54], [58].

In conclusion, our data showed that there was no significance association between sperm DNA damage and C-FET or B-FET outcome after standard IVF or ICSI. Nonetheless, the blastulation rates were significantly higher in the SDF ≤30% group in ICSI cycle. One should expect that different techniques used may bear different results, therefore, it is worthwhile to note that our conclusions based on data with sperm DNA damage measured by the SCD test. Another major limitation of this current study is that sperm DNA fragmentation results are from raw semen and not the sperm fraction used for ART, although the sperm DNA fragmentation index was reportedly a highly stable parameter over a 6 month period [59].
